# Cancer therapy with phytochemicals: evidence from clinical studies

**Published:** 2015

**Authors:** Azar Hosseini, Ahmad Ghorbani

**Affiliations:** 1 *Pharmacological Research Center of Medicinal Plants, School of Medicine, Mashhad University of Medical Sciences, Iran*; 2 *Neurogenic Inflammation Research Centre, Department of Physiology, School of Medicine, Mashhad University of Medical Sciences, Mashhad, Iran*

**Keywords:** *Cancer*, *Phytochemical*, *Plant*, *Tumor*

## Abstract

Cancer is still one of the major causes of mortality in both developing and developed countries. At present, in spite of intensive interventions, a large number of patients suffer from poor prognosis. Therefore, the effort for finding new anticancer agents with better efficacy and lesser side effects has been continued. According to the traditional recommendations and experimental studies, numerous medicinal plants have been reported to have anticancer effect. Also antiproliferative, pro-apoptotic, anti-metastatic and anti-angiogenic effects of several phytochemicals have been shown in *in vitro* experiments or animal studies. However, only a small number have been tested in cancerous patients and limited evidence exists for their clinical effectiveness. Also, regarding some phytochemicals, only beneficial effects on cancer-related symptoms or on quality of life have been reported and no positive results exist for their antitumor actions. This review was focused on the phytochemicals whole beneficial effects on various types of cancer have been supported by clinical trials. Based on the literature review, curcumin, green tea, resveratrol and *Viscum album* were the satisfactory instances of clinical evidence for supporting their anticancer effects. The main findings of these phytochemicals were also summarized and discussed.

## Introduction

Cancer is a growing health problem in both developing and developed countries. According to the recent report of World Health Organization (February, 2014), 8.2 million patients died from cancer in 2012. It has been also estimated that the number of annual cancer cases would have increase from 14 million in 2012 to 22 million within the next two decades (WHO, 2014 ▶). Currently, the main treatments for cancer are chemotherapy, radiotherapy and surgery. Some of the most used chemotherapy drugs include antimetabolites (e.g. methotrexate), DNA-interactive agents (e.g. cisplatin, doxorubicin), antitubulin agents (taxanes), hormones and molecular targeting agents (Nussbaumer et al., 2011 ▶). However, clinical uses of these drugs are accompanied with by several unwanted effects such as hair loss, suppression of bone marrow, drug resistance, gastrointestinal lesions, neurologic dysfunction and cardiac toxicity (Nussbaumer et al., 2011 ▶; Monsuez et al., 2010 ▶; Dropcho, 2011). Moreover, even with the current intensive interventions, a large number of patients suffer from poor prognosis. Therefore, the search for new anticancer agents with better efficacy and lesser side effects has been continued. Natural compounds are good sources for the development of new remedies for different diseases. Experimentally, several medicinal plants and herbal ingredients have been reported to have anticancer effects (Sharma et al., 2011 ▶; Teiten et al., 2013 ▶; Tan et al., 2011 ▶). Also, a number of phytochemicals isolated from medicinal plants have been shown to decrease cell proliferation, induce apoptosis, retard metastasis and inhibit angiogenesis (Hajzadeh et al., 2006 ▶; Tavakkol-Afshari et al., 2006 ▶; Mortazavian et al., 2012 ▶; Mortazavian and Ghorbani, 2012 ▶; Sadeghnia et al., 2014 ▶; Shu et al., 2010 ▶; Tan et al., 2011 ▶). Currently, some of these plant-derived compounds are widely used for chemotherapy of cancerous patients. For example, taxol analogues, vinca alkaloids (vincristine, vinblastine), and podophyllotoxin analogues have played an important role in treatment of such patients (Saklani and Kutty, 2008 ▶). This review presents an update of published data on medicinal plants and herbal ingredients that their anticancer effects have been supported by clinical trials (Table 1).

**Table 1:** Phytochemicals clinically tested in cancerous patients

**Phytochemical**	**Patients**	**Study Design**	**Intervention**	**Effect**	**Reference**
*Allium sativum*	Patients with inoperable colorectal, liver, or pancreatic cancer	Randomized double-blind placebo-controlled trial	4 garlic capsules per day for 12 weeks, 4 capsules contained 500 mg of aged garlic extract	Increase of number and activity of natural-killer cells	Ishikawa et al., 2006
	Patients with colorectal adenomas	Randomized double-blind trial	3 capsules twice a day for 12 months, 6 capsules containing the equivalent of 2.4 ml of garlic	Suppress of size and number of colon adenomas	Tanaka et al., 2006
Camptothecin	Patients with refractory cancers	Phase I clinical trial	Camptothecin: 3 weeks on drug with a 1-week rest; Nitrocamptothecin: 5 consecutive days with a 2-day rest period	Both compounds lead to tumor regression in a number of patients with breast, prostate and melanoma cancers	Natelson et al., 1996
	Patients with primary or metastatic lung cancer	Prospective phase I/II clinical trial	6.7-26.6 µg/kg/day aerosolized liposomal nitrocamptothecin for 5 consecutive days/week for 1-6 weeks followed by 2 weeks of rest	Stabilization occurred in 3 patients with primary lung cancer and partial remissions were happened in 2 patients with uterine cancer	Verschraegen et al., 2004
	Patients with metastatic colorectal cancer.	Phase II clinical trial	Intravenous infusion of 100 mg/m^2^ of CPT-11, a new camptothecin derivative, weekly, or as 150 mg/m^2^ every 2 weeks	Partial response was observed in 17 of 63 patients (6 of 40 patients with liver metastases and 11 of 28 patients with lung metastases)	Shimada et al., 1993
Curcumin	Patients with urinary bladder cancer, uterine cervical neoplasm, or intestinal metaplasia	Prospective phase I/II clinical trial	500 mg/day, orally, for 3 month	Histologic improvement in 1 out of 2 patients with bladder cancer, 1 out of 6 patients with intestinal metaplasia and 1 out of 4 patients with uterine cervical neoplasm	Cheng et al., 2001
	Patients with advanced pancreatic cancer	Nonrandomized open-label phase II trial	8 g/day curcumin, orally, for one mouth	Among 21 patients, 1 had stable disease for >18 months and 1 had tumor regression	Dhillon et al., 2008
Green tea	Patients with high-grade prostate intraepithelial neoplasia	Double-blind placebo-controlled trial	600 mg/day green tea catechins, orally, for one year	After 1 year, the incidence of tumor development was 3% and 30% in treated and control men, respectively; quality of life improved	Bettuzzi et al., 2006
	Patients with histologically confirmed adenocarcinoma of the prostate	Case-control study	Usual tea consumption	The prostate cancer risk declined with increasing frequency, duration and quantity of green tea consumption green tea	Jian et al., 2004
	Patients with esophageal cancer	Case-control study	Usual tea consumption	consumption was associated with reduced risk of esophageal cancer	Gao et al., 1994
	Patients with colon, rectum and pancreas cancer	Case-control study	Regular, non-regular and high tea consumption	An inverse association with each cancer was observed with increasing amount of green tea consumption	Ji et al., 1997
	Patients with hormone refractory prostate cancer	Self-control study	250 mg twice daily for 2 months	Among 15 patients, 9 subjects had progressive disease within 2 months and 6 patients developed progressive disease after additional 1 to 4 months of therapy	Choan et al., 2005
	Patients with hormone refractory prostate cancer	Self-control study	250 mg twice daily for 2 months	Among 15 patients, 9 subjects had progressive disease within 2 months and 6 patients developed progressive disease after additional 1 to 4 months of therapy	Choan et al., 2005
*Panax ginseng*	Patients with cancer of uterine, ovary, rectum, stomach, etc	Randomized double-blind placebo controlled pilot trial	3000 mg/day of the heat-processed ginseng for 12 weeks	Improvement of mental and physical functioning	Kim et al., 2006
	Patients with stage III gastric cancer	Not stated	4.5 g/day of ginseng powder for 6 months; patients were followed up for 4.5 years	Significant reduction of cancer recurrence and restoration of CD3 and CD4 levels to the initial preoperative values	Suh et al., 2002

**Literature Review and Method**

The clinical studies included in this review were identified through a literature review conducted on Google Scholar, Medline and Science Direct using the key terms *cancer, clinical, plants*, *herbs* and* patients*. Only clinical studies investigating the anticancer effect of phytochemicals on cancerous patients were included in the manuscript. Regarding some phytochemicals (e.g. *Aloe vera* and *Withania somnifera*), only beneficial effects on cancer-related symptoms (e.g. fatigue, pain, vomiting, and anorexia) or on quality of life have been reported and no positive results existed for their antitumor actions (Biswal et al., 2013 ▶; Su et al., 2004 ▶). Reports of these clinical trials have not been included. Also, for some phytochemicals, the number of trials supporting their antitumor actions on cancerous patients was not more than the number of those reporting no anticancer effects. These phytochemicals (e.g *Punica granatum*) were excluded because their efficacies need to be investigated further.

**Anticancer** **Phytochemicals**

***Allium sativum***

*Allium sativum* (garlic) has been reported to have a number of medicinal attributes including antidiabetic, hypolipidemic, antimicrobial, antihypertensive and anticancer effects (Hajzadeh et al., 2006 ▶; Tavakkol-Afshari et al., 2006 ▶; ^Ghorbani, 2013a ▶^). Epidemiologic studies have suggested that the consumption of garlic is associated with a protective effect against gastrointestinal cancers (Fleischauer et al., 2000 ▶). Recently, it has been demonstrated that garlic intake suppresses the progression of colorectal adenomas in humans (Tanaka et al., 2006 ▶). Also, a randomized double-blind placebo-controlled trial showed that administering of garlic increases the number and activity of natural-killer cells in the patients with advanced cancer of the digestive system (Ishikawa et al., 2006 ▶). Because increase of natural-killer cell is associated with a favorable tumor outcome, garlic may prevent death due to cancer (Coca et al., 1997). 

**Camptothecin**

Camptothecin is a natural alkaloid which can be extracted from several plant species including *Canzptotheca acirminata *and *Mappia foetida* (Natelson et al., 1996). It is a potent antitumor phytochemical that targets topoisomerase I, an enzyme involved in the relaxation of DNA supercoils (Natelson et al., 1996; Li et al., 2006 ▶). Several derivatives of camptothecin have been synthesized and are in clinic trials. In a phase I clinical study, 20-(S)-camptothecin and 20-(S)-9-nitrocamptothecin were administrated to 52 and 29 patients with refractory cancers, respectively. Both compounds showed antitumor effects in a number of patients with breast cancer (disappearance of liver mass), prostate cancer (fall in the rapidly rising PSA level) and melanoma (regression of skin tumor nodules). Also partial responses were observed in a number of patients with cholangiocarcinoma (reduction in widespread liver metastases), breast carcinoma (disappearance of skin metastases) and ovarian carcinoma (Natelson et al., 1996). Another study demonstrated that the administration of aerosolized liposomal 9-nitrocamptothecin caused positive results (stabilization and partial remissions) in some patients with advanced pulmonary malignancies (Verschraegen et al., 2004 ▶). Moreover, it was reported that CPT-11 (a new camptothecin derivative) could induce an antitumor effect against metastatic colorectal cancer (Shimada et al., 1993 ▶).

**Curcumin**

Curcumin is a yellow polyphenol (diferuloylmethane) derived from the rhizome of turmeric (*curcuma longa* Linn). Extensive experimental and clinical works over the past decade have addressed its therapeutic effects against several diseases including diabetes, cardiovascular disease, arthritis, gastrointestinal ulcers, nephropathy and hepatic disorders. The beneficial actions of curcumin are related to its anti-inflammatory, antioxidant and cytoprotective properties (Gupta et al., 2013 ▶; Alinejad et al., 2013 ▶; Noorafshan and Ashkani-Esfahani, 2013 ▶). Moreover, it has been represented that curcumin possess anticancer effects through its multiple actions on mutagenesis, cell cycle regulation, apoptosis, oncogene expression and metastasis (Wilken et al., 2011 ▶). Different stages of cancer including initiation, promotion and progression can be affected by curcumin. Cheng et al. (2001) ▶ showed that this compound improved histologic parameters in 1 out of 2 patients with resected bladder cancer, 1 out of 6 patients with intestinal metaplasia of the stomach and 1 out of 4 patients with uterine cervical intraepithelial neoplasm. In a nonrandomized open-label study, 25 patients with pancreatic cancer were enrolled in an oral curcumin administration. Among them, two patients showed clinical responses: one had stable disease for >18 months and the other had tumor regression (Dhillon et al., 2008 ▶). In a study by Sharma et al. (2001) ▶, ingestion of curcumin was accompanied by a significant decrease in lymphocytic glutathione S-transferase (GST) activity. The GSTs are a family of phase II detoxification enzymes and have been shown to be involved in the development of resistance to chemotherapy drugs (Townsend and Tew, 2003 ▶). The antitumor action of curcumin is mediated via its antiproliferative effect in multiple cancers, inhibitory action on transcription factors and downstream gene products, modulatory effect on growth factor receptors and cell adhesion molecules involved in angiogenesis, tumor growth and metastasis (Wilken et al., 2011 ▶).

**Green tea**

Green tea is a popular beverage which is consumed worldwide especially in Asia, Europe and North America. Several biological properties have been reported for this beverage which include anti-inflammatory, anti-arthritic, antimicrobial, antioxidative, neuroprotective, antidiabetic, antiangiogenesis and anticancer effects. Green tea contains proteins, amino acids, carbohydrates, minerals, lipids, vitamins and volatile compounds (Chacko et al., 2010 ▶; ^Ghorbani, 2013b ▶^). Recent studies have identified that catechins, a type of polyphenols, are active constituents responsible for the most of the biological properties of green tea (Bettuzzi et al. 2006 ▶; Chacko et al., 2010 ▶). In a double-blind placebo-controlled study, Bettuzzi et al. (2006) ▶ showed that green tea catechins were safe and effective for treating premalignant prostate cancer. On the other hand, results of studies of Jatoi et al. (2003) and Choan et al. (2005) showed that green tea has minimal clinical activity against prostate cancer. Besides, case-control studies support the protective effect of green tea against the prostate, esophageal, colon, rectum and pancreatic cancers (Gao et al., 1994 ▶; Ji et al., 1997; Jian et al., 2004). Also, preliminary results reported by Shanafelt et al.(2006) ▶ suggested that green tea may be useful in the patients with low grade B-cell malignancies. 

***Panax ginseng***

Roots of ginseng have been medicinally used for thousands of years in Asia. *Panax quinquefolius *(American ginseng), *Panax japonicus *(Japanese ginseng) and *Panax ginseng *(Korean or Chinese ginseng) are of the most commonly used ginsengs worldwide (Ghorbani, 2013b). According to the clinical trials, *P. ginseng* decreases the incidence of cancer and shows beneficial effects in cancerous patients. Case-control studies have revealed that fresh sliced ginseng, the juice, or tea decreases the risk of most types of cancers including pharynx, larynx, esophagus, stomach, colorectal, pancreas, liver, lung and ovary (Yun and Choi, 1990; Yun and Choi, 1995 ▶). In a cohort of 1455 breast cancer patients, it was found that consumption of ginseng before cancer diagnosis was associated with increased overall survival rate (Cui et al., 2006 ▶). Regarding the quality of life, although Bao et al. (2012) ▶ did not find any positive results, Cui et al., (2006) ▶ reported that use of ginseng increased this parameter. Further, a randomized placebo- controlled trial showed that *P. ginseng* improved some aspects of mental and physical functioning in patients with gynaecologic or hepatobiliary cancer (Kim et al., 2006 ▶).

**Rsveratrol**

Resveratrol (*trans*-3, 4′, 5-tri-hydroxystilbene) is a phytoalexin found in the skin of grapes, peanuts, a variety of berries and some other fruits. It is known to induce potent antioxidant and anti-inflammatory effects and inhibit the proliferation of a variety of cancer cells (Athar et al., 2007 ▶; Smoliga et al., 2011 ▶). The antiproliferative effect of resveratrol is mediated through the inhibition of several transcription factors, up-regulation of p53, caspases and Bax, and down-regulation of survivin, cyclins and Bcl-2 (Aggrawal et al., 2004 ▶). Increase of Bax/Bcl-2 ratio and up-regulation of caspases lead to apoptosis (Ghorbani et al., 2014). The beneficial effects of resveratrol against cancer have been shown in all the stages of carcinogenesis; initiation, promotion and progression (Smoliga et al., 2011 ▶). Nguyen et al. (2009) ▶ showed that although resveratrol-containing grape powder can-not suppress the Wnt pathway in colon cancer, it could inhibit Wnt target gene expression in normal colonic mucosa of the patients with colorectal cancer. This suggests that resveratrol has a beneficial effect in the initiation and metastasis of colon cancer because Wnts and their downstream effectors regulate the processes involved in tumor initiation, tumor growth, cell death and metastasis (Anastas and Moon, 2013 ▶). Consistent with this suggestion, it has been shown that the administration of resveratrol to patients with colorectal adenocarcinoma reduces tumor cell proliferation (Patel et al., 2010 ▶). Also, in the patients with colorectal cancer and hepatic metastases, resveratrol increases caspase-3 in malignant hepatic tissue compared with the tissue from the placebo-treated patients (Howells et al., 2011 ▶). The results suggest that consumption of resveratrol induces anticarcinogenic effects in human gastrointestinal tract.

***Rhus verniciflua***

*Rhus verniciflua* (the current name is* Toxicodendron vernicifluum*) is native to East Asia and cultivated in some regions of Japan, Korea and China. This plant is known as "lacquer tree" and has been used for many years in traditional Korean medicine for its antioxidant, antimicrobial, anti-inflammatory and anticancer properties (Jianhua et al., 2010 ▶; Kim et al., 2013 ▶; Lee et al., 2010b). Experimental studies have shown that ﬂavonoids from Rhus verniciﬂua have anti-proliferative and apoptotic activities on various tumor cell lines including human lymphoma, breast cancer, osteosarcoma and transformed hepatoma cells (Son et al., 2005 ▶; Jang et al., 2005 ▶; Samoszuk et al., 2005 ▶ and Lee et al., 2004 ▶). A Korean single-center study reported that standardized *R. verniciflua *extract positively affects overall survival rate in the patients with advanced or metastatic pancreatic cancer and in cases with metastatic colorectal cancer (Lee et al., 2009a; Lee et al., 2011). The positive effect on survival rate and disease progression time have been also reported for patients with advanced non-small cell lung cancer, which arise the possibility that *R. verniciflua *has synergetic effects with concurrent therapy agents and may be appropriate in the patients that chemotherapy is not possible (Lee et al., 2009b; Cheon et al., 2011 ▶). Recently, Lee et al. (2010a) reported the successful treatment of two patients with metastatic renal cell carcinoma by allergen-removed *R. verniciflua *extract. The beneficial actions of *R. verniciflua* should be considered alongside its allergenic effect which may induce severe contact dermatitis in sensitive individuals (Lee et al., 2009b).

***Viscum album***

*Viscum album* is a species of mistletoe (Santalaceae) and is commonly  known as European mistletoe. Phytochemical preparations of mistletoe are among the most frequently prescribed complementary and alternative therapies for cancer in Europe (Kienle et al., 2003 ▶). Until 2003, 23 clinical trials were done to investigate efficacy of mistletoe in cancer. Among them, 19 trials reported positive results on survival, quality of life, or tumor remission in cancerous patients (Kienle et al., 2003 ▶). Recent works have also shown that a standardized aqueous extract of *V. album* can decrease side-effects of chemotherapy and improve quality of life in cancerous patients (Piao et al., 2004 ▶; Semiglasov et al., 2004 ▶). Researches have continued on *V. album*-prepared phytochemicals such as Iscador and aviscumine and recent findings are shown in Table 1. Iscador is a lacto-fermented extract of fresh sap of *V. album*. It has been shown that Iscador increases survival time and self-regulation and decreases suppression of natural killer cell activity in patients with carcinoma of the colon, rectum, stomach, breast and bronchus (Grossarth-Maticek et al., 2001 ▶; Schink et al., 2007 ▶). Besides, Kleeberg et al. (2004) ▶ reported that Iscador had no clinical benefit in high-risk melanoma patients. Regarding aviscumine, preclinical studies have demonstrated immunomodulatory effects which may induce positive effect on tumor stabilization (Schöffski et al., 2004 ▶; Zwierzina et al., 2011 ▶). Yet, further clinical trials are needed to establish the therapeutic value of aviscumine in the management of diabetic cancer.

**Other anticancer phytochemicals**

Some other phytochemicals, in addition to the above-mentioned ones have been found to have beneficial effects on cancerous patients: *Aloe arborescens* (Lissoni et al., 2009 ▶), combretastatins (Stevenson et al., 2003 ▶), *Ganoderma lucidum* (Gao et al., 2003 ▶), *Nigella sativa* (Hagag et al., 2013 ▶), *Panax quinquefolius* (Barton et al., 2010 ▶), *Scutellaria barbata* (Perez et al., 2010 ▶) and tetrahydrocannabinol (Guzman et al., 2006 ▶). However, for each one, only one or two clinical studies from independent authors could be found for support the anticancer effects. Therefore, more clinical works are necessary to confirm their therapeutic values for the cancer management.

## Conclusion

Since cancer is commonly fatal and increasing in the world, there is an urgent need for finding new remedies. Medicinal plants have been always an important source for the discovery of new therapeutics for human diseases. Therefore, this source may be a good candidate for the development of novel anticancer agents. Among the hundreds of plants that have been studied for cancer, only a small number of them pass* in vitro* experiments and animal studies and are under clinical trials. Based our literature search, *Allium sativum*, camptothecin, curcumin, green tea, *Panax ginseng*, resveratrol, *Rhus verniciflua* and *Viscum album* had satisfactory instances of clinical evidence for supporting their anticancer effects. Therefore, it seems that they can be used as a complementary therapeutics along with current chemotherapy drugs against various types of cancer. Although a number of other phytochemicals could be also added to this list, it is better to remain until more clinical trials support their anticancer effect. An overall weakness of cancer phytotherapy is that in most of related studies there are methodological flaws including lack of control or placebo group, small sample sizes and short duration of trial. Therefore, for many of phytochemicals, it is too early to draw conclusion for their anticancer actions. Moreover, much remains to be learned about pharmacokinetics, drug interactions, ideal dosages, long-term safety and adverse effects of phytochemicals proposed for cancer treatment. On the other hand, fortunately molecular mechanisms responsible for anticancer effect of several medicinal plants have been revealed by *in vitro* studies. These mechanisms include antioxidation, carcinogen inactivation, antiproliferation, cell cycle arrest, induction of apoptosis, and inhibition of angiogenesis or a combination of these mechanisms (Chahar et al., 2011 ▶).

It is believed that herbal preparations containing multiple phytochemicals may have greater effects than the same phytochemical taken separately. Based on this belief, combinations of the anticancer phytochemicals may have more effect and yield more potent therapeutic agents for cancer. The initial results are encouraging (de la Taille et al., 1999 ▶; Zhuang et al., 2009 ▶) and upcoming clinical trials on this topic are particularly warranted.

## Conflict of interest

The authors declare that there is no conflict of interests regarding the publication of this paper.
